# Novel therapeutic strategies for rare mutations in non-small cell lung cancer

**DOI:** 10.1038/s41598-024-61087-2

**Published:** 2024-05-05

**Authors:** Qitao Gou, Qiheng Gou, Xiaochuan Gan, Yuxin Xie

**Affiliations:** 1 Department of Radiation Oncology and Department of Head & Neck Oncology, Cancer Center, West China Hospital, Sichuan University, Chengdu, China; 2 Department of Oncology, The First Affiliated Hospital of Chongqing Medical University, Chongqing, China; 3 Department of Medical Oncology of Cancer Center, West China Hospital, Sichuan University, Chengdu, China

**Keywords:** Non-small cell lung cancer, Target therapy, Oncologic driver, Rare mutations, Cancer, Oncology

## Abstract

Lung cancer is still the leading cause of cancer-related mortality. Over the past two decades, the management of non-small cell lung cancer (NSCLC) has undergone a significant revolution. Since the first identification of activating mutations in the epidermal growth factor receptor (*EGFR*) gene in 2004, several genetic aberrations, such as anaplastic lymphoma kinase rearrangements (*ALK*), neurotrophic tropomyosin receptor kinase (*NTRK*) and hepatocyte growth factor receptor (*MET*), have been found. With the development of gene sequencing technology, the development of targeted drugs for rare mutations, such as multikinase inhibitors, has provided new strategies for treating lung cancer patients with rare mutations. Patients who harbor this type of oncologic driver might acquire a greater survival benefit from the use of targeted therapy than from the use of chemotherapy and immunotherapy. To date, more new agents and regimens can achieve satisfactory results in patients with NSCLC. In this review, we focus on recent advances and highlight the new approval of molecular targeted therapy for NSCLC patients with rare oncologic drivers.

## Introduction

Lung cancer is a widespread form of cancer that affects many individuals around the world. While the treatment of advanced lung cancer has undergone significant improvements in the past two decades, the global cancer statistics continue to reveal a grim reality. Over 1.8 million patients succumb to lung cancer each year, with incidence rates continuing to rise in developing countries. In both the USA and China, lung cancer will remain the leading cause of cancer-related mortality in 2022. This troubling outcome is largely attributed to the fact that more than half of all lung cancer patients are diagnosed with advanced stage lung cancer. Non-small cell lung cancer (NSCLC) is the most prevalent form of lung cancer, accounting for approximately 85% of newly diagnosed lung cancer cases^1,2^.

The median overall survival (OS) is approximately 4–5 months for metastatic NSCLC patients who only receive supportive care. However, when patients received supportive care combined with induction platinum-based chemotherapy (cisplatin/paclitaxel; carboplatin/paclitaxel; cisplatin/docetaxel; carboplatin/paclitaxel), the median OS improved to 8–12 months. Over the past few decades, several trials have compared different chemotherapy regimens, but OS has improved slightly^3,4^. Researchers believe that research on the efficacy of chemotherapy has stagnated.

In 2002, a phase III trial compared four regimens (cisplatin/paclitaxel; cisplatin/gemcitabine; cisplatin/docetaxel; carboplatin/paclitaxel) that were platinum-based doublets in first-line metastatic NSCLC, but the trial demonstrated that there was no difference in these regimens^5^. When researchers first identified activating mutations of epidermal growth factor receptor (*EGFR*) in 2004, gefitinib also demonstrated a significantly greater survival benefit than chemotherapy in EGFR-positive patients simultaneously^6,7^. Therefore, the development of targeted therapy and the identification of specific driver mutations are major advances in the treatment of metastatic NSCLC. Moreover, a phase III trial compared gefitinib and platinum-based doublets, and the results indicated that targeted therapy is superior to carboplatin-paclitaxel as an initial treatment for *EGFR*-positive NSCLC patients^8^. Due to the discovery of biomarkers and the effectiveness of targeted therapy, the existing histopathological classification of lung cancer has been completely reconstructed. For instance, in stages III and IV, the use of gefitinib has less toxicity in patient with EGFR-positive status than dose chemotherapy^9^. This also warrants a separate listing in the National Comprehensive Cancer Network (NCCN) guidelines of targeted therapy as the first-line treatment for patients with driver gene-positive status among those receiving palliative care^10^. Additionally, researchers have paid more attention to this type of rare subgroup. Biomarkers of anaplastic lymphoma kinase rearrangement (*ALK*), recombinant C-Ros Oncogene 1 (*ROS1*), v-raf murine sarcoma viral oncogene homolog B1 (*BRAF*), and human epidermal growth factor receptor 2 (*HER-2*), biomarkers have been discovered in recent decades^11–14^. Due to the incidence rate of some targets in lung cancer (≤ 5%), these new potential oncogenic drivers are divided into rare subgroups. In addition, corresponding agents have been developed simultaneously. Some clinical trials have shown that the response rate and OS are significantly increased in patients who receive targeted therapy^15–17^.

In the past decade, tremendous changes have taken place in the treatment of advanced NSCLC. Molecular profiles can be divided into different subgroups to help determine treatment plans, and enable the patient to receive personalized treatment. Patients who harbor oncologic driver mutations can receive FDA-approved targeted therapy as first-line therapy. However, most of these novel oncologic drivers do not have clear therapeutic algorithms; in some cases, some agents that can be used for the desired indication have not been approved by the FDA. Whereas, the relatively low frequency of these genetic aberrations makes it difficult to conduct large-scale randomized clinical trials. Furthermore, some oncologic driver mutations that can cause drug resistance, such as the EGFR T790M mutation and G810 solvent-front rearranged during transfection (*RET*) mutants, also cause difficulties for researchers^18,19^.

In this review, we summarize some novel and emerging actionable oncogenic drivers in NSCLC and focus on the main clinical challenges in patients who harbor rare oncologic drivers. These new agents and regimens can provide patients more choices and greater survival benefits.

## Epidermal growth factor receptor (*EGFR*) exon 20 insertion

The EGFR gene is expressed in 12% of white NSCLC patients and more than 50% of Asian NSCLC patients^20–23^. Since researchers identified the *EGFR* gene and developed gefitinib in 2004, three generations of EGFR inhibitors have been developed. These EGFR inhibitors have changed therapeutic algorithms and provided patients with significant survival benefits. *EGFR* exon 20 insertion (ex20ins) mutations are the third most common EGFR mutation subtype, and they are found in nearly 4% of *EGFR*-mutant NSCLC patients. It mainly occurs in Asians, women, nonsmokers and populations with adenocarcinoma. However, it is different from other common *EGFR*-mutant NSCLCs because it is not sensitive to first- or second-generation EGFR-tyrosine kinase inhibitors (EGFR-TKIs)^24–26^.

Previous studies have indicated that alterations in the drug-binding pocket of exon 20 lead to reduced drug action. 3D modeling revealed that *EGFR ex20ins* have two structures: a C-helix domain and a loop following the C-helix domain. In this structure, *EGFR ex20ins* mutations result in a shift of the phosphate-binding loop (P-loop) into the drug-binding pocket and increased affinity for ATP. This change can reduce the binding of first-generation inhibitors and lead to drug resistance^27,28^. Therefore, other agents, such as osimertinib, poziotinib and mobocertinib, have been designed to treat patients with the *EGFR* ex20ins mutation and have showed promising results in recently updated data (Table 1).

**Table 1:** Summary of targeted therapy in the principal clinical trial of *EGFR ex20ins*.

Drug	Trial name	Phase	Dose	Patients	Main results	Status
Osimertinib	ECOG-ACRIN 5162 (NCT03191149)	II	160 mg qd	20 ≥ 2 L	ORR: 25% DCR: 85% mPFS: 9.7 m mDOR: 5.7 m	Recruiting
	POSITION20 (NL6705)	II	160 mg qd	25 ≥ 1 L	ORR: 28% mPFS: 6.8 m mOS: 15.2 m	Active
Poziotinib	ZENITH20 (NCT03318939) cohort 1	II	16 mg qd	88 ≥ 2 L	ORR: 14.8% DCR: 68.7% mDOR: 7.4 m mPFS: 4.2 m	Terminated
	NCT03066206	II	16 mg qd	50 ≥ 1 L	ORR: 32% (total)/46% (near loop)/0% (far loop) DCR: 85% mPFS: 5.5 m	Active
Mobocertinib (TAK-788)	EXCLAIM (NCT02716116)	I/II	160 mg qd	114 ≥ 2 L	ORR: 28% DCR: 78% mDOR: 17.5 m mOS: 24.0 m mPFS: 7.3 m	Active
				86 1 L	ORR: 25% DCR: 76% mDOR: 17.3 m mPFS: 7.3 m	
Zipalertinib (CLN-081; TAS6417)	NCT04036682	I/II	30 mg to 150 mg bid	70 ≥ 2 L	PR: 25 (36%) SD: 34 (49%)	Recruiting
Amivantamab (JNJ372)	CHRYSALIS (NCT02609776)	I	1050 mg (< 80 kg) or 1400 mg (≥ 80 kg) qw (1-4w), q2w (≥ 5w)	81 ≥ 2 L	ORR: 40% mDOR: 11 m mPFS: 8.3 m mOS: 22.8 m	Active
	PAPILLON; NCT04538664	III	1400 mg (< 80 kg) or 1750 mg (≥ 80 kg) qw (1-4w), q3w (≥ 7w)	308 ≥ 1 L	ORR: 73% DCR: 92% mDOR:9.7 m mPFS: 11.4 m	Active
Sunvozertinib (DZD9008)	WU-KONG1(NCT03974022) And WU-KONG15(NCT05559645)	II	200 mg qd 300 mg qd	28 ≥ 2 L	ORR: 71.4% (200 mg, 68.4%; 300 mg, 77.8%)	Recruiting
	WU-KONG6 (NCT05668988)	II	300 mg qd	97 ≥ 1 L	ORR: 60.8% DCR: 87.6%	Recruiting
Furmonertinib (Alflutinib/AST2818)	FAVOUR 1 (NCT04858958)	I/II	160 mg qd	25 ≥ 2 L	ORR: 40.9% DCR: 90.9% mPFS:5.8 m	Recruiting
			240 mg qd	24 ≥ 2 L	ORR: 50% DCR: 95.5% mPFS: 7.0 m	
			240 mg qd	30 1 L	ORR: 69% DCR: 96.6% mPFS: 10.7 m	

*ORR* overall response rate, *m* month, *mDOR* median duration of response, *mPFS* median progression-free survival, *mOS* median overall survival, *DCR* disease control rate, *PR* partial response, *SD* stable response, *qd* once daily, *bid* twice daily, *qw* once week, *1 L* first-line, *2 L* second-line.

### Osimertinib

Osimertinib, a third-generation EGFR-TKI, was approved by the Food and Drug Administration (FDA) as the first agent for acquired drug resistance after EGFR-TKI treatment. Some studies have shown that the IC50 of osimertinib for *EGFR ex20ins* is 10-100-fold greater than that for other *EGFR* mutations^29,30^. Interestingly, a retrospective Chinese study enrolled 6 patients with ex20ins + NSCLC who received osimertinib 80 mg once daily, and showed promising results. However, more studies have reported disappointing evidence of the efficacy of osimertinib 80 mg once daily^25,31–33^. In contrast, trials with high doses of osimertinib (160 mg) have shown promising antitumor activity. A phase II (NCT03191149) study of patients who received osimertinib 160 mg per day and showed an overall response rate (ORR) of 25% and a DCR of 85%. The median progression-free survival (mPFS) and median duration of response (DOR) were 9.7 months and 5.7 months, respectively^34^. Another study (POSITION20) also obtained similar results^35^. These studies showed that osimertinib cannot be routinely recommended (80 mg daily) for patients with NSCLC harboring ex20ins. A higher dosing scheme study should be developed.

### Poziotinib

Poziotinib is an oral EGFR inhibitor that can inhibit *EGFR* mutations and *HER-2* alternations. Researchers have demonstrated that poziotinib has better effects than other TKIs in vitro with *EGFR ex20ins*. Furthermore, 3D modeling also indicated that poziotinib has small terminal and substituent linkers that make it more flexible and reasonable than other EGFR-TKIs for binding the ex20ins receptor^36^. Several corresponding studies also showed a similar result to that of osimertinib, with an ORR of nearly 30%, DCR of approximately 85% and a manageable safety profile^37,38^. Interestingly, researchers have also classified loops following the C-helix domain as near- and far-loop insertions and found that the sensitivity of pozidinib is highly dependent on the insertion position of the insertion in the loop following the C-helix domain. The results indicated that near-loop insertions are more sensitive than far ring insertions, with ORRs of 46% and 0%, respectively^38^. Unfortunately, a phase 2 clinical trial (ZENITH20; NCT03318939) showed an ORR of 14.8% and a DCR of 68.7%. However, due to the primary endpoint was not met and there was a high incidence of grade 3 side effects, especially rash (28%) and diarrhea (26%)^39^. Moreover, in the ZENITH20-2 trial, rash and diarrhea were the predominant grade 3 treatment-related adverse events (TRAEs), occurring in 78.9% of all patients, and leading to the discontinuation of poziotinib in 13% of cases^37^. The management of adverse effects has proven to be challenging, resulting in a significant proportion of patients receiving suboptimal doses and durations of therapy. At present, this study has terminated.

### Mobocertinib (TAK-788; AP32788)

Mobocertinib, a novel irreversible EGFR-TKI, can selectively inhibit the *EGFR* and *HER-2 ex20ins* mutations. A corresponding phase II study (EXCLAIM, NCT02716116) showed relatively promising results (ORR: 43%, mDOR: 14 months; mPFS: 7.3 months)^40^. Subsequently, an extension cohort in which patients who previously received platinum-based chemotherapy and who were evaluated for morbidity had an ORR of 28% and DCR of 78%, and side effects were well tolerated^41^. In July 2021, based on the ORR and long-term survival benefit of by the EXCLAIM trial, mobocertinib was accepted by the Center for Drug Evaluation of the National Medical Products Administration of China, and the FDA accelerated the approval of the application of mobocertinib for *EGFR ex20ins*-positive NSCLC patients who had previously received platinum-based chemotherapy in September 2021^27,42^. Additionally, an ongoing phase 3 randomized trial (EXCLAM-2; NCT04129502) evaluated the efficacy of combination treatment with mobiocertinib and platinum-doublet chemotherapy as first-line treatments among patients with treatment-naïve advanced NSCLC whose tumors harbor *EGFR ex20ins* mutations. This study is the first clinical study to prospectively evaluate the efficacy of *EGFR ex20ins* targeted therapy compared with that of the current first-line standard therapy, and the results are eagerly anticipated. A phase III randomized study (EXCLAIM-2; NCT04129502) did not indicate a promising outcome with mobocertinib monotherapy in comparison to chemotherapy as the first-line treatment choice for this patient group. As a result, an announcement was made regarding the voluntary withdrawal of mobocertinib in the United States, which had previously received accelerated approval^43^.

### Zipalertinib (CLN-081; TAS6417)

Zipalertinib is a novel irreversible EGFR-TKI that can inhibit *EGFR ex20ins*. In the 2022 American Society of Clinical Oncology (ASCO) annual meeting, a relevant study (NCT04036682) showed significant antitumor activity, and the results indicated that 66 (94%) patients achieved partial response (PR) or stable disease (SD). Moreover, two patients had observable brain metastasis regression, which makes researchers exciting^44^. The FDA has recently granted breakthrough therapy designation for the treatment of locally advanced non-small cell lung cancer (NSCLC) in patients with *ex20ins* mutations who have previously undergone platinum-based chemotherapy^45^.

### Amivantamab (JNJ-372)

Amivantamab is a bispecific monoclonal antibody that can protect against the oncologic drivers of *EGFR* and *MET*^46^. Preclinical studies have demonstrated that amivantamab has more antitumor activity than other EGFR agents and can suppress cell lines harboring *EGFR ex20ins* mutations^47,48^. Due to these promising preclinical findings, the CHRYSALIS trial (NCT02609776) exhibited favorable antitumor activity (ORR: 40%, mOS: 22.8 months)^49^. On May 21, 2021, the FDA approved amivantamab for treating patients with locally advanced or metastatic NSCLC harboring the *EGFR ex20ins* mutation whose disease progressed on or after platinum-based chemotherapy^50,51^. Based on the breakthrough effect of amivantamab, in March 2020, amivantamab was awarded FDA breakthrough therapy designation, marking the first instance of such recognition for an *ex20ins* targeted therapy. Subsequently, in May 2021, another milestone was achieved by becoming the first *ex20ins* targeted therapy to receive FDA approval^50^. Besides, another study of amivantamab plus chemotherapy versus chemotherapy alone as a first-line treatment in patients with *ex20ins* also showed promising results (PAPILLON; NCT04538664). In this study, the combination arm exhibited a markedly greater response rate than did the chemotherapy arm (ORR: 73% vs 43%). Additionally, the combination arm also showed a faster response time to respond and durable efficacy. It should be mentioned that it also has a great effect on brain metastases. In terestingly, although the incidence of side effects in the amivantamab group was greater than that in the chemotherapy group, there were no differences between the two groups in terms of serious side effects^52^.

### Sunvozertinib (DZD9008)

Sunvozertinib (DZD9008) is an oral, irreversible fourth-generation tyrosine kinase inhibitor (TKI)^53^. The phase 2 trial WU-KONG6 enrolled 97 Chinese patients with EGFR Ex20Ins in the post-platinum setting. It showed a notable ORR of 60.8%, and achieved a DCR of 87.6%^54^. Moreover, the results from phase 1/2 WU-KONG1 (NCT03974022) and phase 2 WUKONG15 (NCT05559645) trials, also showed a significant result, with ORRs of up to 68.4% and 77.8% for doses of 200 mg and 300 mg, respectively^55^. An ongoing phase 3 study, WU-KONG28, compared the efficacy of sunvozertinib (300 mg) with that of platinum-pemetrexed in the first-line treatment of patients with *ex20ins* mutations. Despite ongoing investigations, the breakthrough therapy designation by the US FDA has already been granted based on promising outcomes in patients with a history of platinum-based therapy^56^.

### Furmonertinib (Alflutinib/AST2818)

Furmonertinib is a fourth-generation EGFR-TKI that has a similar structure to that of osimertinib^27^. The phase 1b FAVOUR 1 trial (NCT04858958) has currently enrolled 79 patients in three arms: the treatment-naive group (n = 30) received a daily dose of 240 mg, and the previously treated group received 240 mg (n = 24) and 160 mg (n = 25). The ORRs were 69.0%, 50.0%, and 40.9% respectively, with DCRs of 96.6%, 95.5% and 90.9% observed in all subgroups. The mPFS was 10.7 months for the treatment-naïve group and 7.0 months and 5.8 months for the previously treated groups receiving 240 mg and 160 mg, respectively. Of interest, an antitumor response was observed in patients with both near and far-loop EGFR Exon20ins mutations^57^. Although it has a similar structure to osimertinib, compared to POSITION20, the efficacy of furmonertinib is better than that of osimertinib. Moreover, further studies are required to comprehensively evaluate the efficacy of this medication in this patient subgroup. Multiple ongoing trials in China (NCT05466149) and the United States (NCT05364073, FURMO-002) aim to address this need^58^.

### Another clinical trial

Recently, updared data on osimertinib, poziotinib, CLN-081, JNJ-372, mobocertinib, sunvozertinib and furmonertinib are promising. Other agents also have also been developed, and corresponding trials are ongoing. Tuxobertinib (BDTX-189) is an EGFR-TKI that was proven by a preclinical study, and a relevant phase II study is ongoing (NCT04209465). Other related TKIs are currently under evaluation in clinical trials, including FWD1509 (NCT05068024), and JMT101 (NCT04448379), are currently under evaluation in clinical trials^27^.

## Mesenchymal-epithelial transition (*MET*)

*MET* oncologic drivers, also known as hepatocyte growth factor receptor (HGFR), are present in approximately 5% of patients with nonsquamous NSCLC, which is comparable to the frequency of ALK fusion^59^. It is located on human chromosome 7q21-q31 with 21 exons and 20 introns and encodes receptor tyrosine kinase for hepatocyte growth factor (HGF). Then, downstream signaling pathways, including RAS/ERK/MAPK and PI3K/AKT pathways, are activated when HGF specifically binds to the MET receptor. To date, several aberrations in Met have been found, including protein overexpression (15–70%), amplification (2–5%) and *MET* exon 14 (*METex14*) skipping mutations (3–4%)^59,60^. Additionally, aberrant MET occurs in 10–20% of NSCLC patients with EGFR mutations who previously received EGFR-TKIs.

In 2011, an NSCLC patient with *MET* amplification received crizotinib and achieved a rapid and durable response^61^. To date, there are two types of MET-related TKIs: nonselective MET-TKIs and selective MET-TKIs. Among the nonselective MET-TKIs, crizotinib was the first nonselective MET inhibitor to obtain FDA approval. A related study (Profile 1001, NCT00585195) showed a promising result in patients with *METex14* mutations and a high gene copy number (GCN) in MET amplification. Similar results were also shown in other trials, such as AcSè (NCT02034981) and METROS (NCT02499614)^62–65^. Other agents, such as capmatinib, tepotinib, and savolitinib, have also provided favorable data in patients harboring *METex14* skipping mutations and *MET* amplification with high GCN (Tables 2, 3)^66^. With the rapid expansion of data on MET inhibitors, more agents and clinical trials have been developed in recent years. Besides, most of this evidence is based on phase I and II studies, so phase III studies are warranted to confirm the efficacy and safety of MET inhibitors in various cancers.

**Table 2:** Summary of targeted therapy in principal clinical trials of *MET* mutations.

Drug	Trial	Phase	Patients	MET aberrant type	Dose	Main results	Status
Crizotinib	PROFILE 1001 (NCT00585195)	I	65	MET exon 14 and 16–19 mutation	250 mg bid in continuous 28-d cycles	ORR: 32% mPFS: 7.3 m mOS: 20.5 m mDOR: 9.1 m	Completed
	AcSé (NCT02034981)	II	28	MET exon 14 skipping mutation	250 mg bid in continuous 28-d cycles	ORR: 10.7% mPFS: 2.4 m mOS: 8.1 m	Complete
Capmatinib	GEOMETRY mono-1 (NCT02414139)	II	69 ≥ 2 L	MET exon 14 skipping mutation	400 mg bid	ORR: 41% mDOR: 9.7 m mPFS: 5.4 m	Active
			28 1 L			ORR: 68% mDOR: 12.6 m mPFS: 12.4 m	
	NCT01324479	I	4	MET exon 14 skipping mutation	400 mg/600 mg bid	ORR: 75% DCR: 100% (CR: 1; PR: 2; SD: 1)	Completed
Tepotinib	VISION (NCT02864992)	I	69 1 L	MET exon 14 skipping mutation	500 mg qd	ORR: 44.9% DCR: 68.1 m mDOR: 10.8 m mPFS: 8.5 m	Active
			83 ≥ 2 L			ORR: 44.6% DCR: 72.3% mDOR: 11.1 m mPFS: 10.9 m	
Savolitinib	NCT02897479	II	70 ≥ 2 L	MET exon 14 skipping mutation	600 mg qd; 400 mg qd	IRC-ORR:49.2% DCR: 82.9% mPFS: 6.8 m mDOR: 8.3 m	Active
Glumetinib (SCC244)	GLORY (NCT04270591)	II	69 ≥ 2 L	MET exon 14 skipping mutation	300 mg qd in continuous 21-d cycles	ORR: 60.9% mDOR: 8.2 m mPFS: 7.6 m	Recruiting
Amivantamab (JNJ-372)	CHRYSALIS (NCT02609776)	I	43	MET exon 14 skipping mutation	1050 mg (< 80 kg) or 1400 mg (≥ 80 kg) qw (w1) and q2w (≥ 2w)	ORR: 33% 6 m-DOR: 67% mPFS: 6.7 m	Active

*ORR* overall response rate, *m* month, *mDOR* median duration of response, *mPFS* median progression-free survival, *mOS* median overall survival, *DCR* disease control rate, *IRC* independent review center, *qd* once daily, *bid* twice daily, *qw* once week, *w* week, *d* day, *1 L* first-line, *2 L* second-line.

**Table 3:** Summary of principal target therapy in the principal clinical trial of *MET* amplification and overexpression.

Drug	Trial	Phase	Therapy line	Patients	Treatment arm	Main results	Status
Crizotinib	Profile 1001 study (NCT00585195)	I	≥ 1 L	37	Crizotinib 250 mg bid	ORR: MET/CEP7 category: low (≥ 1.8– ≤ 2.2) 33.3%; medium (> 2.2– < 5) 14.3%; high (≥ 5) 40.0% mPFS: low 1.8 m; medium 1.9 m; high 6.7 m	Completed
	METROS (NCT02499614)	II	≥ 1 L	26	Crizotinib 250 mg bid	ORR: 27% mPFS: 4.4 m mOS: 5.4 m	Unknown
Capmatinib	GEOMETRY mono-1(NCT02414139)	II	≥ 1 L	69	Capmatinib 400 mg bid	GCN ≥ 10: ORR: 100% (1 L); 45.5% (2/3 L) mDOR: 8.2 m (1 L) and 8.3 m (2/3 L)	Active
	NCT01610336	II	2 L	100	Capmatinib 400 mg bid + Gefitinib 250 mg qd	ORR: 29% (total); 47% (GCN ≥ 6); 32% (IHC 3 +) DCR: 73% mDOR: 5.6 m	Completed
	NCT01324479	I	≥ 1 L	15	Capmatinib 400 mg/600 mg bid	GCN ≥ 6: ORR: 47% mPFS: 9.3 m	Completed
Tepotinib	INSIGH (NCT01982955)	Ib/II	2 L	18	Tepotinib 500 mg qd + Gefitinib 250 mg qd vs. Platinum-based chemotherapy	Total: mPFS: 4.9 m vs. 4.4 m mOS: 17.3 m vs. 18.7 m IHC3 + : mPFS: 8.3 m vs. 4.4 m mOS: 37.3 m vs. 17.9 m amplification: mPFS: 16.6 m vs. 4.2 m mOS: 37.3 m vs. 13.1 m	Completed
Savolitinib	TATTON (NCT02143466)	Ib	≥ 1 L	B1: Prior 3G EGFR-TKI: 69 B2: No prior 3G EGFR-TKI, T790M (-): 51 B3: No prior 3G EGFR-TKI, T790M ( +): 18 D: treatment naïve, T790M (-): 42	osimertinib 80 mg + savolitinib 600/300 mg (Part B1, B2, B3) osimertinib 80 mg + savolitinib 300 mg (Part D)	B1: ORR: 33% mPFS: 5.5 m mDOR: 9.5 m mOS: 30.3 m B2: ORR: 65% mPFS: 9.1 m mDOR: 10.7 m mOS: 18.8 m B3: ORR: 67% mPFS: 11.1 m mDOR: 11.0 m D: ORR: 62% mPFS: 9.0 m mDOR: 9.7 m	Active
Cabozantinib	NCI 9303 II (NCT01866410)	II	2 L	37	Cabozantinib 40 mg qd + Erlotinib 150 mg qd	ORR = 10.8% mPFS = 3.6 m mOS = 13.1 m	Completed

*ORR* overall response rate, *m* month, *mDOR* median duration of response, *mPFS* median progression-free survival, *mOS* median overall survival, *DCR* disease control rate, *IRC* independent review center, *GCN* gene copy number, *IHC* immunohistochemistry, *qd* once daily, *bid* twice daily, *qw* once week, *w* week, *d* day, *1 L* first-line, *2 L* second-line, *3G* third generation; ( +): positive; (–): negative.

### Capmatinib

Capmatinib is a small molecule highly selective MET-related TKI that belongs to MET-class Ib inhibitors^67^. A previous study showed that capmatinib plus chemotherapy can achieve favorable results in *EGFR*-mutated, *MET*-amplified NSCLC^68^. Then, the GEOMETRY mono-1 study (NCT02414139) and another study (NCT01324479) showed that capmatinib has a meaningful benefit in patients with MET-dysregulated NSCLC^10,49^. In particular, in the high copy number subgroup and *METex14* subgroup, these cohorts showed a high ORR (75%) or DCR (100%) and a considerable survival benefit^69,70^. According to these trials, capmatinib was approved by the FDA for first- and subsequent-line treatment of patients with MET-dysregulated advanced NSCLC. Moreover, because of the successful results of the GEOMETRY mono-1 study, the next phase III GeoMETry-III trial (NCT04427072) is currently evaluating capmatinib compared to docetaxel in pretreated NSCLC patients. Moreover, capmatinib has antitumor activity in crizotinib-pretreated patients with MET-altered NSCLC, although it has modest efficacy (n = 15; ORR: 10%; DCR: 80%)^71^. In this study, patients did not respond well to capmatinib despite disease stabilization. Researchers should focus on class II MET TKIs (merestinib and glesatinib), which do not rely on interactions with the activation loop, and some preclinical trials have also proven this scenario (Tables 2, 3)^72^.

### Tepotinib

Tepotinib is a highly selective, type I MET-TKI for the treatment of non-small cell lung cancer harboring MET alterations. It was approved by the Japanese Ministry of Health, Labor and Welfare and the FDA in March 2020 and February 2021, respectively^73^. Recently, updated data from the phase II VISION trial (NCT02864992) showed promising results in 152 NSCLC patients with a *METex14* skipping mutation. Both the treatment-naïve cohort (n = 69) and previously treated cohort (n = 83) had similar ORRs (44.9% vs 44.6%) and DCRs (68.1% vs 72.3%). Additionally, 13 of 15 patients with central nervous system (CNS) metastases achieved intracranial disease control, which is indicative of strong blood‒brain barrier penetration ability^74^. Furthermore, tepotinib has shown antitumor activity against EGFR-mutated NSCLC patients with *MET* amplification and high *MET* expression. The INSIGHT trial (NCT01982955) included 18 eligible patients and demonstrated that the tepotinib plus gefitinib group had significantly better results than the chemotherapy group in patients with high *MET* overexpression and *MET* amplification^75^. It promoted the emergence of the INSIGHT 2 clinical trial (NCT03940703), which demonstrated that tepotinib plus osimertinib promoted in *MET*-amplified NSCLC after patients experienced osimertinib resistance (Tables 2, 3)^76^.

### Savolitinib

Savolitinib is an oral type Ib MET-TKI that has received approval in China for the treatment of metastatic NSCLC with *METex14* mutation in patients who have progressed after or who are unable to tolerate platinum-based chemotherapy in June 2021^77^. A phase II clinical trial (NCT02897479) enrolled 70 patients with positive pulmonary sarcomatoid carcinoma or other NSCLC subtypes and showed satisfactory results. Independent review center (IRC) reported an ORR of 49.2% and a DCR of 82.9%^78^. Furthermore, savolitinib has demonstrated efficacy in overcoming acquired *MET*-mediated osimertinib resistance. In the TATTON study (NCT02143466), which enrolled 144 patients in part B and 42 patients in part D, updated data revealed an ORR of 33–67% and 62% and an mPFS of 5.5–11.1 and 9.0 months, respectively^79^. These studies have demonstrated that savolitinib has promising antitumor activity and a durable response in patients harboring *MET*-related dysregulation (Tables 2, 3).

### Amivantamab (JNJ-372)

Amivantamab, a bispecific antibody targeting *EGFR* and *MET*, has been shown to disrupt *EGFR* and *MET* signaling functions by ligand blocking and receptor degradation^80^. Previously, at the 2022 ASCO annual meeting, researchers reported data from the ongoing phase I CHRYSALIS (NCT02609776) study. In the MET-2 cohort, 43 patients with NSCLC harboring *METex14* mutations had a favorable outcomes, with an ORR of 33% and a clinical benefit rate of 58.3%. The mPFS was 6.7 months (Table 2)^81^.

### Other agents for MET alteration

Although MET-selective TKIs, such as capmatinib, tepotinib, and savolitinib, have become the new standard of care for treating NSCLC, the combination of MET-TKIs and EGFR-TKIs (osimertinib plus savolitinib, tepotinib plus gefitinib) may be a potential solution for preventing MET-driven EGFR-TKI resistance. Other drugs have been developed and shown promising results in both preclinical and updated data. Glumetinib (SCC244), a highly selective class II MET inhibitor, has shown promising effects in patients with *MET* alterations. An ongoing clinical trial (GLORY; NCT04270591) has reported an ORR of 60.9%, an mDOR of 8.2 months, and an mPFS of 7.6 months by a blinded independent review committee (BIRC). These data show the excellent efficacy of glumetinib in NSCLC patients harboring *METex14* mutations. Furthermore, it also exhibited intracranial antitumor activity with a median intracranial tumor shrinkage of 57%^82^. Cabozantinib is a type of TKI that targets multiple receptors, such as VEGFR, MET, and AXL. However, a previous study (NCI 9303 II; NCT01866410) showed that the combination of cabozantinib and erlotinib had limited effects on in patients with MET amplification. Another clinical trial (NCT03911193) is currently underway, that includes patients with MET alterations^83^. Merestinib (LY2801653) is a multikinase inhibitor that can inhibit *MET*, *ROS1*, and *AXL*, and an ongoing phase II clinical trial (NCT02920996) involving NSCLC patients harboring *METex14* skipping mutations^84^. Additionally, S49076 is a multikinase inhibitor that can inhibit *MET*, *AXL*, and *FGFR1-3* oncologic drivers, and a relevant pivotal phase I/II study (EudraCT: 2015-00264631) is ongoing. Recent results have shown that 2 patients harboring *MET* dysregulation responded to treatment with S49076^85^. In addition, other agents, such as glesatinib (MGCD265), vebrreltinib (APL-101) and telisotuzumab vedotin (Teliso-V), have also been developed in recent years, and relevant clinical trials are currently underway (Table 2)^86,87^.

## V-Raf murine sarcoma viral oncogene homolog (*BRAF*) V600E

The V-Raf murine sarcoma viral oncogene homolog (*BRAF*) gene, located on chromosome 7, has been identified as a well-known oncogene. *BRAF* mutations can be found in various tumors, and the most common tumor is melanoma. Approximately 2–4% of NSCLC patients have *BRAF* gene mutations, with the *BRAF V600E* mutation being detected in 50% of these cases patients^60,88,89^. Mutation of the BRAF V600E protein has been found to affect an enzyme that plays a crucial role in the RAS/RAF/MEK/ERK (MAPK/ERK) signaling pathway, which is essential for the proper functioning of cells and is involved in a range of biological processes such as cell growth, differentiation, and survival^90^. Initially, targeted therapy for the *BRAF* V600e mutation relied on vemurafenib or dabrafenib. However, these treatments did not yield favorable results, with response rates of only 42% and 33%, respectively^91,92^. Trametinib, a MEK protein inhibitor regulated by *BRAF*, combined with dabrafenib can be much more effective in NSCLC with the *BRAF V600E* mutation (NCT01336634). Recently, researchers have updated this clinical trial, and research has revealed that the ORR can increase to 63–69%, regardless of previous treatment. The mPFS was 10.8 months, and the 5-year survival rate was 22% in treatment-naïve patients. In patients in the previous system treatment group, the mPFS was 10.2 months, and the 5-year survival rate was 32%^93^. Another clinical trial also showed favorable results, and the ORRs of patients receiving dabrafenib plus trametinib (D + T) as a second-line or above or as a first-line therapy were 73.8% and 82.9%, respectively^94^. Trametinib alone or in combination with other therapies has shown excellent potential and could become a new standard treatment for BRAF V600E-positive advanced NSCLC.

## Rearrangement during transfection (*RET*)

The *RET* gene is a well-known proto-oncogene and was mapped to the long arm of 10q11.2 chromosome 10. The RET protein is a membrane tyrosine kinase receptor. *RET* mutation and *RET* fusion/rearrangements are more likely to occur in thyroid cancer than in lung cancer. Nearly 2% of lung adenocarcinoma patients have *RET* fusion/rearrangement. To date, more than 15 kinds of *RET* fusions or rearrangements have been discovered in NSCLC. The most common *RET* fusion gene in NSCLC is kinesin family member 5B (*KIF5B*)-*RET* (70–90%), and coil-coil domain containing 6 (*CCDC6*)-*RET* (10–25%) is the second most common fusion gene^95^.

### First-generation RET inhibitors

First-generation RET inhibitors are also known as multikinase inhibitors (MKIs). Multiple trials have demonstrated that MKIs, such as vandetanib and cabozantinib, have favorable efficacy in patients with *RET*-altered NSCLC, and these two agents have been approved by the FDA to treat *RET*-altered NSCLC^96,97^. However, some disadvantages, such as adverse events and drug resistance, affect therapeutic efficacy and limit clinical application. Therefore, a second-generation RET inhibitor, a selective RET inhibitor, was developed. Selpercatinib (LOXO-292) and pralsetinib (BLU-667) are representative selective RET inhibitors.

### Second-generation RET inhibitors

Both selpercatinib (LOXO-292) and pralsetinib (BLU-667) are selective RET inhibitors and corresponding pivotal clinical trials have shown significant results. Based on these trials, the *RET* oncologic driver has demonstrated its potential and might become a well-known oncologic driver as well as ALK and ROS1 (Table 4).

**Table 4:** Summary table of second- and third-generation RET inhibitors in recent and ongoing clinical trials.

Drug	Trial name	Phase	Patients	Dose	Main result	CNS	Status
Selpercatinib (LOXO-292)	LIBRETTO-001 (NCT03157128)	I/II	69 1 L	Phase I cohort: 20 mg qd; 20–240 mg bid, Phase II cohort: 160 mg bid	ORR: 84% mDOR: 20.2 m mPFS: 21.9 m	ORR: 85%	Recruiting
			247 ≥ 2 L		ORR: 61% mDOR: 28.6 m mPFS: 24.9 m		
Pralsetinib (BLU-667)	ARROW (NCT03037385)	I/II	27 1 L	400 mg qd	ORR: 70% DCR: 85% mDOR: 9.0 m mPFS: 9.1 m	ORR: 56%	Active
			87 ≥ 2 L		ORR: 61% DCR: 91% mPFS: 17.1 m		
	AcceleRET-Lung (NCT04222972)	III	75 1 L	400 mg qd	ORR: 72% DCR: 100% mPFS: 13.0 m	ORR: 70%	Active
			136 ≥ 2 L		ORR: 59% DCR: 90% mDOR: 22.3 m mPFS: 16.5 m		
TPX-0046	Sword-1 (NCT04161391)	I/II	10 ≥ 1 L	5 mg/kg bid	PR: 1 SD: 3		Terminated
Zeteletinib (BOS-172738)	NCT03780517	I	30 ≥ 1 L	≥ 10 mg qd	ORR: 33%		Completed

*ORR* overall response rate, *m* month, *mDOR* median duration of response, *mPFS* median progression-free survival, *mOS* median overall survival, *DCR* disease control rate, *PR* partial response, *SD* stable response, *qd* once daily, *bid* twice daily, *qw* once week, *w* week, *d* day, *1 L* first-line, *2 L* second-line.

The potency of pralsetinib was ≥ tenfold greater than that of MKIs approved by the FDA. In September 2020, the FDA granted accelerated approval to pralsetinib for adult patients with metastatic *RET* fusion-positive NSCLC. The ARROW study (NCT03037385), a pivotal clinical trial of pralsetinib, recently enrolled 233 patients who were diagnosed with *RET* fusion-positive NSCLC. The cohort included 27 patients who were newly diagnosed with NSCLC and 87 who had previously received platinum-based chemotherapy, and the ORRs were 70% and 61%, respectively^98^. Another clinical trial (NCT04222972) also showed a similar effect after treatment with pralsetinib. Interestingly, pralsetinib treatment resulted in tumor shrinkage in 97% of patients. Additionally, the observed 70% intracranial response rate is particularly surprising. These results demonstrate that pralsetinib is highly effective in treating primary tumors and metastases, including intracranial tumors^99^.

In May 2020, selpercatinib, a new agent that was used to treat *RET*-altered NSCLC, was approved by the FDA. The LIBRETTO-001 clinical trial (NCT03157128) revealed a striking survival benefit for NSCLC patients who were *RET* fusion-positive. Until 2022, the ORR was 61% in 247 patients who had previously been treated with chemotherapy. The median DOR was 28.6 months, and the median PFS was 24.9 months. Moreover, 69 treatment-naïve patients had a better ORR (84%) than those previously treated with chemotherapy, and the updated data reported a median DOR of 20.2 months and a median PFS of 22.0 months. Interestingly, selpercatinib can also cross the blood‒brain barrier and respond to intracranial masses. The intracranial ORR was 85%, and 27% of the patients achieved CR according to the updated data^100^. Another study also demonstrated similar results in patients with NSCLC who had brain metastases^101^. These striking results indicate that selpercatinib is a good therapeutic option for patients with *RET*-altered NSCLC.

### Third-generation inhibitors

Two patients were treated with selpercatinib, and disease progression was observed. This is because the *RET G810R/S/C* solvent front mutation has been acquired and caused drug resistance^18^. Therefore, researchers are currently developing third-generation inhibitors to inhibit additional *RET* mutations that confer resistance to MKIs and second-generation RET inhibitors (Table 4).

TPX-0046 is a famous third-generation inhibitor, and it has strong potency against solvent-front *RET G810* mutants. It has a smaller and more rigid macrocyclic structure than second-generation inhibitors, and this structure can generate a compact type I inhibitor and maintain antitumor activity without drug resistance^102^. Sword-1 (NCT04161391) is a pivotal clinical trial for TPX-0046, and it received favorable data were obtained in the initial study. As of March 2021, preliminary clinical data were collected from 14 evaluable patients with NSCLC and medullary thyroid carcinoma (MTC), which included 5 TKI-naïve patients (3 with NSCLC and 2 with MTC) and 9 previously TKI-treated patients (4 with NSCLC and 2 with MTC). Interestingly, 4 patients in the TKI-naïve group experienced tumor regression, and 3 patients in the TKI-pretreated group experienced tumor shrinkage. Moreover, the majority of treatment-related adverse events (TRAEs) were grade 1 or 2, and no grade 4 or 5 TRAEs were found^103^.

Zeteletinib (BOS-172738; DS-5010) is a small molecule RET inhibitor that shows favorable potency for various *RET* mutations, such as the M918T, V840L and V840M gatekeeper mutations. An ongoing phase I clinical trial (NCT03780517) is pivotal study for zeteletinib, and it exhibited favorable safety and ORR. As of December 2020, the study enrolled 30 NSCLC patients and had an NSCLC ORR of 33% (n = 10/33). Zeteletinib also showed safety for long-term administration. Most patients experienced grade 1–2 adverse events that were deemed unrelated to zeteletinib^104^.

## Human epidermal growth factor receptor 2 (HER-2)

Human epidermal growth factor receptor 2 (*HER-2*), also referred to as *ErbB2*, is a well-known proto-oncogene located on chromosome 17 (17q21). Intriguingly, it is a member of the EGFR family of receptor tyrosine kinases, and is composed of three segments: an extracellular ligand binding domain, an α-helical transmembrane segment, and an intracellular tyrosine kinase domain. Unlike other members of the EGFR family, no natural ligand has been identified for HER-2. Ligand binding to other receptors in the family promotes receptor dimerization, leading to the activation of downstream signaling pathways, such as the PI3K/Akt and Ras/MAPK pathways. In *HER-2*-altered cancer cells, this constitutive activation results in uncontrolled cell growth^105^.

*HER-2* alterations are frequently observed in various cancer types^106–108^. However, *HER-2* aberrations can be identified in small subsets of NSCLC patients. Three HER-2-activating mechanisms have been found in NSCLC: mutation (occurring in 1–4% of cases), amplification ( occurring in 2–5% of cases) and overexpression (occurring in 10–15% of cases)^109^. Among *HER-2* mutations, exon 20 insertions are the most frequent *HER-2* mutations, accounting for 96% of *HER-2* mutations. It is easily and predominantly found in patients who are young and nonsmokers, and there is no correlation between race, sex, lymph node involvement and tumor stage. Additionally, *HER-2* mutations are an independent poor prognostic factor^110^. The ability of amplification and overexpression of *HER-2* to distinguish between amplification and overexpression is still unclear. In addition, a meta-analysis demonstrated that *HER-2* overexpression might be an independent prognostic factor in patients with NSCLC^110–112^. Currently, practitioners use immunohistochemistry (IHC), which categorizes the staining intensity on a scale from 0 to 3 + to determine the Her-2 amplification and overexpression. This system defines IHC 0–1 + as HER2 negative, IHC 2 + as weak to moderate, and IHC 3 + as strong when staining occurs in 10% of tumor cells. Furthermore, there is a significant relationship between HER-2 protein expression, assessed through IHC, and HER-2 gene copy number, as determined by FISH, with many patients showing polysomy rather than true amplification. Consequently, HER-2 2 + or 3 + expression should prompt the addition of FISH analysis to differentiate between these possibilities^113–115^. In addition, many prospective studies have failed to identify an association between the response of patients with an aberrant *HER-2* gene and conventional chemotherapy^112^. These studies urge researchers to develop new agents to treat these types of patients, such as small molecule TKIs and anti-HER-2 antibodies, and bring new hope to patient with this currently incurable disease (Table 5).

**Table 5:** Summary table of principal clinical trials of HER-2 inhibitors.

Drug	Study	Phase	Patients	HER-2 alternation type	Dose	Main results	Status
Tyrosine kinase inhibitor							
Afatinib	NICHE (NCT02369484)	II	13	HER-2 exon 20 mutations	40 mg qd	ORR: 7.7% DCR: 53.8% mPFS: 15.9w mOS: 56w	Completed
Dacomitinib	NCT00818441	II	26	HER-2 alternation	30–45 mg qd	ORR: 12% mPFS: 3 m mOS: 9 m	Completed
Neratinib	SUMMIT (NCT01953926)	II	26	HER-2 mutation	240 mg qd	ORR: 3.8% DCR: 42.3% mPFS: 5.5 m	Completed
Poziotinib	ZENITH20-2 (NCT03318939)	II	90 ≥ 2 L	HER-2 exon 20 mutations	16 mg qd	ORR: 27.8% DCR: 70% mPFS: 5.5 m	Terminated
	NCT03066206	II	30 ≥ 2 L	HER-2 exon 20 mutations	16 mg qd	ORR: 27% mDOR: 5.0 m mPFS: 5.5 m mOS: 15 m	Active
Pyrotinib	NCT02535507	II	15 ≥ 2 L	HER-2 mutation (A775_G776YVMA insertion)	400 mg qd	ORR: 53.3% DCR: 73.3% mPFS: 6.4 m mOS: 12.9 m	Unknown
	NCT02834936	II	60 ≥ 2 L	HER-2 exon 20 mutations	400 mg qd	ORR: 30% DCR: 85% mPFS: 6.9 m mOS: 14.4 m	Unknown
	ChiCTR1800020262	II	27 ≥ 1 L	HER-2 amplification	400 mg qd	ORR: 22.2% mPFS: 6.3 m mOS: 12.5 m	Recruiting
Tarloxotinib	RAIN-701 (NCT03805841)	II	9 ≥ 2 L	HER-2 mutation	150 mg/m2 iv qw	ORR: 22% DCR: 44%	Terminated
Monoclonal antibodies							
Trastuzumab	HOT1303-B (UMIN000012551)	II	10 ≥ 1 L	HER-2 alternation	6 mg/kg q3w	ORR: 0% DCR: 70% mPFS: 5.2 m	Completed
Trastuzumab + pertuzumab/ChT	DRUP (NCT02925234)	II	24 ≥ 2 L	HER-2 exon 20 mutation	pertuzumab: loading dose: 840 mg D1; maintain dose: 420 mg q3w; trastuzumab: loading dose 8 mg/kg; maintain dose: 6 mg/kg q3w;	ORR: 8.3% mPFS: 4 m mOS: 10 m	Recruiting
	MyPathway (NCT02091141)	II	30	HER-2 alternation (mutation = 14; A&O = 16)	pertuzumab: loading dose: 840 mg D1; maintain dose: 420 mg q3w; trastuzumab: loading dose 8 mg/kg; maintain dose: 6 mg/kg q3w;	Mutation: ORR: 21% DCR: 43% A&O: ORR: 13% DCR: 25%	Completed
	IFCT 1703-R2D2 (NCT03845270)	II	45 ≥ 2 L	HER-2 mutation	pertuzumab: loading dose: 840 mg D1; maintain dose: 420 mg q3w; trastuzumab: loading dose 8 mg/kg; maintain dose: 6 mg/kg q3w; docetaxel: 75 mg/m2 q3w	ORR: 29% DCR: 77% mPFS: 6.8 m mDOR: 11 m	Completed
	ECOG 2508	II	53	HER-2 overexpression	trastuzumab: loading dose 4 mg/kg; maintain dose: 2 mg/kg q3w; paclitaxel: 225 mg/m2 q3w; carboplatin: AUC = 6 q3w	ORR: 25% mPFS: 3.3 m mOS: 10.1 m	-
Antibody‒drug conjugates (ADC)							
Trastuzumab-emtansine (T-DM1)	JapicCTI-194620	II	22 ≥ 1 L	HER-2 exon 20 mutation	3.6 mg/kg iv q3w	ORR: 38.1% DCR: 52.4% mDOR: 3.5 m mPFS: 2.8 m mOS: 8.1 m	Unknown
	NCT02289833	II	49 ≥ 2 L	HER-2 overexpression (29 IHC 2 + , 20 IHC 3 +)	3.6 mg/kg iv q3w	IHC 3 + : ORR: 20% mPFS: 2.7 m mOS: 15.3 m IHC 2 + : ORR: 0% mPFS: 2.6 m mOS: 12.2 m	Completed
	UMIN000017709	II	15	HER-2 alternation	3.6 mg/kg iv q3w	ORR: 6.7% mPFS: 2.0 m mOS: 10.9 m	Terminated
Trastuzumab-deruxtecan (DS-8201a)	DESTINY-Lung01 (NCT03505710)	II	91 ≥ 2 L	HER-2 alternation (mutation = 42; overexpression = 49)	6.4 mg/kg q3w	Mutation: ORR: 61.9% DCR: 90.5% mPFS: 14 m overexpression: ORR: 24.5% DCR: 69.4% mPFS: 5.4 m mOS: 11.3 m	Active
	NCT02564900	I	11 ≥ 2 L	HER-2 mutation	6.4 mg/kg q3w	ORR: 72.7% DCR: 90.9% mPFS: 11.3 m mOS: 17.3 m	Complete

*ORR* overall response rate, *m* month, *mDOR* median duration of response, *mPFS* median progression-free survival, *mOS* median overall survival, *DCR* disease control rate, *IHC* immunohistochemistry, *A&O* amplification or overexpression, *ChT* chemotherapy, *qd* once daily, *bid* twice daily, *qw* once week, *w* week; *d* day, *1 L* first-line, *2 L* second-line.

### Small molecule tyrosine kinase inhibitors

Nonselective HER-2 tyrosine kinase inhibitors, including afatinib, dacomitinib, and neratinib, have shown limited results in clinical trials. Afatinib, a pan-Her TKI, was first evaluated for efficacy in patients with *HER-2* mutation-positive solid tumors, including NSCLC. However, in the NICHE clinical trial (NCT02369484), the result was unsatisfactory in a clinical trial (ORR = 7.7%). Only one patient with *HER-2*-mutant NSCLC achieved a PR^116^. Another nonselective pan-Her TKI (dacomitinib, neratinib) also showed unsatisfactory results^117–119^. These clinical trials have led researchers and practitioners to pay more attention to selective HER-2 TKIs (poziotinib, pyrotinib, tarloxotinib, etc.) and design related clinical trials that have shown favorable results.

Recently, novel, more selective pan-HER-2 TKIs, such as poziotinib, pyrotinib, tarloxotinib and mobocertinib, have been developed with the objective of improving outcomes in patients with NSCLC with *HER-2* mutations. Each agent has shown promising antitumor activity in clinical trials.

Poziotinib, a novel, oral, irreversible pan-HER-2 TKI. It has a smaller size and more flexible structure than afatinib, making it more effective than other pan-HER-2 TKIs. Two clinical trials (NCT03318939; NCT03066206) involving participants with *HER-2* mutations who previously received therapy reported highly similar results, with an ORR of nearly 27% and mPFS of 5.5 months^120,121^.

Pyrotinib is another oral, irreversible pan-HER-2 TKI used to treat NSCLC. A previous clinical trial demonstrated that pyrotinib has promising antitumor activity in patients with NSCLC harboring *HER-2 exon 20* mutations^122^. Recently, a single-arm trial including 27 patients with HER-2 amplification showed promising results with an ORR of 22.2% and an mPFS of 6.3 months. Furthermore, pyrotinib can cross the brain‒blood barrier, and patients with brain metastases have an ORR of 40%^123^. However, because the primary endpoint was not met and there was a high incidence rate of grade 3 TRAEs, ZENITH20 trail was terminated.

Other small molecule TKIs, such as tarloxotinib, a pan-HER kinase inhibitor, also showed dramatic treatment efficacy in a patient with the *HER-2 exon 20 p. A775_G776insYVMA* mutation^124^. In addition, a phase II study (NCT03805841) is ongoing and enrolled patients with NSCLC who were pretreated with chemotherapy and harbor EGFR exon 20 insertions or *HER-2* mutations. However, this clinical trial showed a limited effect. Of the nine assessable patients with NSCLC harboring *HER-2* mutations, two patients achieved a PR (ORR of 22%), four patients exhibited SD (DCR of 67%), and most side effects were grade 1–2 (Table 5)^125^. Mobocertinib (TAK-788/AP3278), a next-generation small-molecule oral TKI, is designed to selectively target both EGFR insertions and HER-2 mutations. An ongoing phase I/II study (NCT02716116) is recruiting patients with NSCLC harboring *HER-2 exon 20* alterations^126^.

### Monoclonal antibodies and antibody‒drug conjugates against HER-2

Recently, monoclonal antibodies, such as trastuzumab, and antibody‒drug conjugates (ADCs), which is class of targeted cancer therapies that combine the specificity of monoclonal antibodies (mAbs) with the cytotoxic effects of chemotherapy drugs such as trastuzumab-emtansine and trastuzumab-deruxtecan, which target the HER-2 receptor have shown some results in clinical trials^112,127^.

Trastuzumab is a monoclonal immunoglobulin G1 humanized murine antibody that targets the HER-2 receptor and inhibits its dimerization. It can induce internalization and/or degradation of the receptor, eventually impeding downstream pathways. A previous study demonstrated that monotherapy with trastuzumab in patients with NSCLC harboring *HER-2* gene aberrations has a limited survival benefit^128,129^. In addition, although trastuzumab combined with chemotherapy has shown a promising effects in previous studies, it can be realized that chemotherapy, not trastuzumab, provides survival benefits to patients^130^. More recently, a phase II study (IFCT 1703-R2D2, NCT03845270) also showed the efficacy of the combination of trastuzumab, pertuzumab, and docetaxel in patients with advanced NSCLC harboring *HER-2* mutations who progressed after ≥ 1 platinum-based treatment, and this combination exhibited a feasible result. Forty-five patients were enrolled and showed the ORR was 29%^131^. Another clinical trial (DRUP, NCT02925234; MyPathway, NCT02091141) also showed limited results in patients with *HER-2* alternations who were treated with trastuzumab plus pertuzumab^128,132^. Therefore, trastuzumab shows disappointing results regardless of whether it is used as monotherapy or in combination with chemotherapy.

Trastuzumab-emtansine (T-DM1) is a novel ADC composed of trastuzumab that targets the HER-2 receptor. Past research has shown favorable results in patients harboring *HER-2* mutations^133^. In a recent update, 22 eligible patients were included in this study; the ORR was 38.1%, and the DCR was 52.4%. Moreover, the drug was well tolerated and had manageable side effects^134^. In patients harboring overexpression or amplification, T-DM1 presented a limited result. ORR was found to be 20% in the IHC 3 + group and 0% in the IHC 2 + group^135^.

Trastuzumab-deruxtecan (T-DXd; DS-8201a) is also a novel ADC similar to T-DM1. Recently, updated results from DESTINY-Lung01 (NCT03505710) showed a significant result in 91 patients with *HER-2* mutations who received T-DXd. The results showed an ORR of 55%, an mDOR of 9.3 months, and an mPFS of 17.8 months. Safety is generally acceptable, but it is important to monitor for interstitial pneumonia (ILD)^136^. Another clinical trial also showed significant results^137^. In the HER-2-overexpressing NSCLC cohort. Although the results were not as impressive as those in cohort 2, they were still promising. The ORR was 24.5%, the mDoR was 6 months, and the mPFS was 5.4 months. Interestingly, the response rates were not based on HER-2 IHC expression levels, with an ORR of 20.0% versus 25.6% in IHC3 + and IHC2 + patients, respectively^138^. In August 2022, the FDA granted accelerated approval to T-DXd for patients with unresectable or metastatic NSCLC whose tumors harbored *HER-2* mutations^139^.

Therefore, both T-DM1 and T-DXd have shown promising results in patients with HER-2 mutations and IHC 3 + NSCLC but have limited efficacy in those with IHC 2 + NSCLC. Additionally, patients with HER-2-positive tumors are more likely to have brain metastasis. In the future, combining ADCs with irreversible TKIs, such as pyrotinib, may become a trend, because TKIs can not only penetrate the blood–brain barrier but also increase the antitumor activity. In a preclinical study, researchers demonstrated that combining T-DM1 with a pan-HER irreversible inhibitor such as neratinib enhanced receptor ubiquitination and subsequent internalization of HER-2-ADC complexes, resulting in potent antitumor activity^140^. Furthermore, in the future, patients with IHC 2 + may present more refined subgroups, such as gastric carcinoma with IHC 2 + /fluorescence in situ hybridization (FISH) + , and these subgroups might receive a survival benefit from targeted therapy.

## Neurotrophic tyrosine kinase (NTRK)

Alterations in neurotrophic tyrosine kinase (*NTRK*) genes (*NTRK1, NTRK2, and NTRK3*) are rare alterations in NSCLC, accounting for less than 1% of NSCLC cases^141^. Regarding the NTRK mechanism, the *NTRK* gene encodes the TrkA, TrkB, and TrkC transmembrane glycoproteins, which work with nerve growth factor (NGF), BDNF, neurotrophin-3 (NT-3), and NT-4 to support the development and function of the nervous system. *NTRK* gene fusions lead to the overexpression of Trk proteins and the activation of downstream signaling pathways, such as RAS/MAPK, PI3K/AKT, and PLC-γ pathways, resulting in cancer cell transformation, proliferation, and survival^142^. Although *NTRK* mutations, splice variants, and deletions can occur in certain types of tumor cells, these genetic alterations are generally not responsive to targeted therapy. Therefore, our article will concentrate on *NTRK* fusions, which are genetic changes that have been associated with a favorable response to NTRK-targeted therapy. To date, more than 80 known fusion partners have been identified, and the most frequently detected fusions are ETS Variant Transcription Factor 6 (*ETV6*)-*NTRK3* and echinoderm microtubule associated protein like 4 (*EML4*)-*NTRK3*^59,143^.

The first-generation TRK inhibitors entrectinib and larotrectinib were approved by the FDA due to their impressive results in phase I/II trials^144^. These drugs are designed to target ROS1 and ALK and have shown high response rates in clinical trials. Both entrectinib and larotrectinib yield a high response rate and are well tolerated with few adverse events in previous studies^89,145–148^. At the 2022 ASCO annual meeting, the updated data on larotrectinib also reported a similar result. Among 15 evaluable patients who received larotrectinib, the ORR was 73%, the mPFS was 1.8 months and the mOS was 40.7 months, and the side effects were mainly grade 1–2^149^. Therefore, entrectinib and larotrectinib yield high ORRs and favorable survival benefits for patients, making *NTRK* a major therapeutic target.

The key distinction between them is their ability to penetrate the CNS, with entrectinib being more effective at crossing the blood‒brain barrier^150^. This has been demonstrated in its successful treatment of patients with brain metastases. In a clinical trial, 12 patients with brain metastatic *NTRK* fusion-positive solid tumors received entrectinib, resulting in 6 patients who achieved a PR and 4 with SD. Nonetheless, entrectinib has been associated with several CNS-related adverse effects, such as dizziness^145^.

TRK inhibitors showed favorable effects. However, as with many targeted cancer therapies, the number of cases of tumor-acquired drug resistance to targeted therapy has increased in recent years. Therefore, the next generation of TRK inhibitors, such as taletrectinib (DS-6051b), selirectinib (LOXO-195), and repotrectinib (TPX-0005), are currently undergoing evaluation in ongoing phase I/II clinical trials, offering more hope for patients harboring NTRK alternations^60,144^.

## Fibroblast growth factor receptor (FGFR)

Fibroblast growth factor receptors (FGFRs) are a family of receptor tyrosine kinases that play critical roles in numerous biological processes. The *FGFR* gene family consists of four members (FGFR1-4) that encode structurally similar transmembrane proteins^151,152^. Each FGFR contains an extracellular ligand-binding domain, a transmembrane domain, and an intracellular kinase domain. The binding of ligands, primarily fibroblast growth factors, to the extracellular domain of FGFRs leads to receptor dimerization and autophosphorylation of the intracellular domain. This causes a cascade of downstream signaling pathways, including the RAS/MAPK and PI3K/AKT pathways, and regulates cellular processes such as cell proliferation, differentiation, migration, and survival^152^.

Dysregulated *FGFR* genes include gene involved in amplification, mutation and fusion^152^. According to recent studies, *FGFR* alterations have been found to be more frequent in squamous cell histology (22%) than in adenocarcinomas (3%)^152^. According to a comprehensive genomic profiling study of 26,054 NSCLC specimens, *FGFR* fusions were found to occur in 0.2% of NSCLC patients, with squamous cell histology accounting for 0.59% and adenocarcinoma accounting for approximately 0.12%^152,153^. These mutations are primarily detected in patients without other driver mutations or in a subset of patients as a mechanism of acquired resistance^110^. Recently, the TCGA database showed that 3% of squamous-cell lung cancer (SqCLC) samples have mutations in at least one of the *FGFR2* and *FGFR3* genes. These mutations are mostly *FGFR2* (W290C and S320C) and *FGFR3* (R248C and S249C) in the extracellular domain of the gene and *FGFR2* (K660E and K660N) in the kinase domain. Furthermore, the occurrence probability of *FGFR* gene fusion in SqCLC is approximately 2%-3.5%, with the most frequent sequence being *FGFR3-TACC*^154^.

Nonselective FGFR TKIs, such as ponatinib, dovitinib and pazopanib, have shown promising effects in NSCLC patients. However, toxicity is an important problem for nonselective FGFR inhibitors. Due to the occurrence of severe adverse events, clinical trials are frequently terminated. Therefore, researchers need to develop more effective and selective agents for cancers with *FGFR* aberrations (Table 6)^155,156^.

**Table 6:** Summary table of principal clinical trials of selective FGFR inhibitors.

Agent	Trial	Phase	Patients	FGFR alternation type	Dose	Main results	Status
AZD4547	SWOG S1400D (NCT02965378)	II	27 ≥ 2 L	FGFR alternation	80 mg bid	ORR: 7% mPFS: 2.7 m mOS: 7.5 m	Completed
GSK3052230 (FP-1039) + ChT	NCT01868022	Ib	29 ≥ 1 L	FGFR amplification	GSK3052230: 10/15/20 mg/kg qw ChT: Paclitaxel: 200 mg/m2; Docetaxel: 75 mg/m2; Carboplatin: AUC = 6	ORR: 47% mPFS: 5.5 m	Completed
Rogaratinib (BAY1163877)	SAKK 19/18 (NCT03762122)	II	15 ≥ 2 L	FGFR overexpression	600 mg bid	SD: 7 PD: 5 mPFS: 1.6 m mOS: 3.5 m	Terminated

*ORR* overall response rate, m month, *mDOR* median duration of response, *mPFS* median progression-free survival, *mOS* median overall survival, *SD* stable disease, *PD* progressive disease, *ChT* chemotherapy, *bid* twice daily, *qw* once week, *1L* first line, *2L* second line.

The next generation of FGFR inhibitors are selective FGFR TKIs. Most of the TKIs in this class, such as AZD4547 and GSK3052230, inhibit FGFR1-3. Additionally, some pan-FGFR inhibitors, such as erdafitinib and rogaratinib, can also inhibit FGFR 1–4. Currently, numerous selective FGFR inhibitors are being evaluated in clinical trials^156^.

AZD4547 is an oral ATP-competitive FGFR1-3 inhibitor and has a lower level of activity against *FGFR 4*, *IGF1R*, *MARKs* and *KDR*^157^. The SWOG S1400D trial (NCT02965378) was the first phase II trial to evaluate the efficacy of AZD4547. In 2019, the updated results enrolled 27 response-evaluable patients in the evaluable group who had various *FGFR* alterations were inculded. Although the safety profile was tolerable, this compound exhibited minimal activity in this predominantly *FGFR*-amplified cohort, with an ORR of 7%, mPFS of 2.7 months and mOS of 7.5 months^158^.

GSK3052230 (FP-1039) is a soluble fusion protein that acts as an FGFR ligand trap consisting of the extracellular domain of FGF receptor 1 (FGFR1) fused with the Fc region of IgG1 and subsequently inhibits tumor growth and angiogenesis^159^. The corresponding phase Ib study included 20 patients with metastatic or recurrent SqCLC harboring *FGFR1* gene amplification who received GSK3052230 and chemotherapy. Treatment with GSK3052230 was well tolerated and had favorable antitumor activity (ORR: 47%, mPFS: 5.5 months)^160^.

Rogaratinib (BAY1163877) is an oral pan-FGFR inhibitor that has shown encouraging results in preclinical trials, including for lung cancers harboring *FGFR* alterations^161^. Previous data showed a 5.6% ORR^162^. Recently, a phase II clinical trial (NCT03762122) included 15 patients with *FGFR* alterations. This trial showed that rogaratinib has only limited efficacy in patients with SqCLC harboring *FGFR* overexpression (SD: 7; PD: 5; mPFS: 1.6 months). However, approximately 40% of patients experienced grade ≥ 3 TRAEs. Due to the high incidence of TRAEs and limited efficacy, this clinical trial has been terminated^163^.

In summary, the majority of clinical trials and drugs currently available are still in the research and development stage, and recent data have shown limited effects. Therefore, new drugs should be developed.

## Other rare gene aberrations in lung cancer

Other rare gene aberrations have also been found in NSCLC, and some oncologic drivers have also shown excellent clinical efficacy. For example, the anaplastic lymphoma kinase (*ALK*) receptor gene is located on chromosome 2p23 and encodes a tyrosine kinase receptor. *ALK* fusions have been identified in approximately 5% of NSCLC patients, with the most common fusion partner being echinoderm microtubule-associated protein-like 4 (*EML4*)^164^. Crizotinib, the first-generation ALK inhibitor, was approved by the FDA in 2011 for the treatment of *ALK*-positive NSCLC^165^. However, second- or third-generation ALK inhibitors, such as ceritinib, alectinib, brigatinib, and lorlatinib, have been developed to overcome drug resistance to crizotinib and have shown favorable results in clinical trials^166–169^. Recently, fourth-generation ALK-TKIs have also shown promising results in preclinical studies. For example, NVL-655 showed antitumor activity and the ability to penetrate the blood‒brain barrier in vivo. In addition, it can also against solvent front drug-resistant mutations, a kind of genetic changes in the active site of proteins, particularly enzymes, which can render drugs ineffective by altering the binding shape or properties of site and caused drug-resistance, such as *G1202R*, *G1202R* + *L1196M*, and *G1202R* + *G1269A*^170^. On June 9th, 2022, a phase 1/2 clinical trial (ALKOVE-1; NCT05384626) is ongoing and includes patients with solid tumors harboring *ALK* aberrations, including NSCLC, and patients are receiving NVL-655. Recently, As of August 8^th^, 2023, in a cohort of 51 NSCLC patients, 20 patients achieved a PR, with an ORR of 39%. Additionally, 34 patients continued NVL-655 treatment, with an mDOR of 3.4 months. Notably, in patients with baseline brain metastases (n = 29), the ORR was 52%. Interestingly, the ORR was 54% in patients with ALK-resistant mutations (n = 28)^170^. TPX-0131, a next-generation ALK inhibitor, also exhibited preclinical potency against both wild-type *ALK* and a wide range of compound mutations, such as G1202R + L1198F, L1196M + L1198F, and G1202R + C1156F^171^. Recently, a relevant clinical trial (FORGE-1; NCT04849273) is ongoing. In the 2022 American Society of Clinical Oncology (ASCO) meeting, SAF-189 s, a next-generation ALK inhibitor, achieved excellent results and was well tolerated in patients with advanced, *ALK*-positive NSCLC, with a DCR of 100% in both the ALK inhibitor group and the ALK inhibitor-naïve group. Furthermore, it also showed excellent intracranial penetration in both groups (NCT04237805)^172^. Other ALK-TKIs, including entrectinib, repotrectinib (TPX-0005) and gilteritinib, are under preclinical or clinical investigation^164,173–175^.

The *C-Ros 1* oncogene of the receptor tyrosine kinase (*ROS1*) gene, similar to the *ALK*-positive gene, is also called the a “diamond mutation” because it has a highly homologous structure to the ALK protein and thus has a similar oncogenic characteristics^89^. It was discovered in the 1980s and was first reported in lung cancer in 2007 by Rikova et al.^176^. *ROS1* rearrangements are found in 0.9–2.6% of NSCLC cases, and patients with *ROS1*-positive NSCLC tend to be younger, never-smokers, and diagnosed with adenocarcinoma. ROS-1 rearrangements are considered a driver mutation in NSCLC and are mutually exclusive with other driver mutations, such as *EGFR* and *ALK* rearrangements^177^. Crizotinib was the first agent approved by the FDA for metastatic *ROS1*-positive NSCLC in March 2016. The pivotal phase I PROFILE 1001 trial (NCT00585195) showed excellent results with an ORR of 72% and a DCR of 90%. However, the emergence of drug resistance, including the G2032R point mutation, was observed. In addition, crizotinib also has poor intracranial penetration. Other agents, such as entrectinib, lorlatinib, ceritinib, cabozantinib and brigatinib, have been developed and overcome the disadvantage of weak penetration ability of the blood‒brain barrier^89,178^. Repotrectinib, a next-generation ROS1/TRK and ALK inhibitor, showed good tolerability and significant antitumor activity in a phase II study (TRIDENT-1; NCT03093116). The ORRs were 91% and 57% in treatment-naïve patients and patients who received previous treatment, respectively. It also exhibited excellent intracranial activity, with a 100% intracranial ORR in TKI-naïve patients and a 75% ORR in patients with 1 prior TKI^179^. Taletrectinib (DS-6051B) is a next-generation TKI that targets both ROS1 and NTRK. It also exhibits impressive brain-penetrant ability and activity against the *ROS1 G2032R* resistance mutation. In a phase II clinical study (NCT04395677), 61 eligible patients were enrolled, and the results showed 90% ORR and 95% DCR in patients with crizotinib-naïve tumors. In addition, in the crizotinib-naïve group, the ORR and DCR were 47.6% and 76.2, respectively. Moreover, it also showed excellent intracranial penetration^180^. Another relative phase II study (TRUST-II; NCT04919811) is ongoing^181^. Compared with crizotiib, ensartinib (X-396) is a TKI with tenfold greater in vitro activity against *ALK* than crizotinib. A recent clinical trial (NCT03608007) demonstrated the promising efficacy of ensartinib in *ROS1*-positive NSCLCs with an ORR of 27%. More interestingly, 75% of patients with CNS diseasesachieved disease control^182^.

Genetic rearrangements of neuregulin-1 (*NRG1*) were identified as a recently discovered oncogene driver of NSCLC that was initially reported in 2014. It has been observed in approximately 0.2–0.5% of unselected NSCLC patients^60^. To date, a study evaluated 21,858 tumor specimens from various solid tumors, with the most prevalent variant being *CD74-NRG1* (29%), while *AT1P1-NRG1* (10%) and *SDC4-NRG1* (7%) were the second and third most common^183^. Interestingly, the rearrangement of *NRG1* in solid tumors leads to aberrations in the downstream HER-2/HER-3 signaling pathway. Therefore, a previous study showed that patients harboring *NRG*1 fusion-positive tumors who received afatinib had disappointing results^184^. However, this study provided researchers with treatment ideas. Recently, GSK2849330, an agent that can inhibit the *HER-3* oncologic driver, showed antitumor activity in a patient expressing the *CD74-NRG1* gene fusion (NCT01966445)^185^. Furthermore, zenocutuzumab (MCLA-128) can inhibit HER-2/HER-3 to mediate *NRG1* signaling in tumor cells. A relevant phase II basket trial (NCT02912949) is ongoing^186^. Seribantumab is an anti-HER3 inhibitor that has shown antitumor activity in preclinical models. The relevant clinical trial (CRESTONE; NCT04383210) enrolled patients with *NRG1* fusion-positive solid tumors, including NSCLC^187^.

## Conclusions

**Figure 1:**
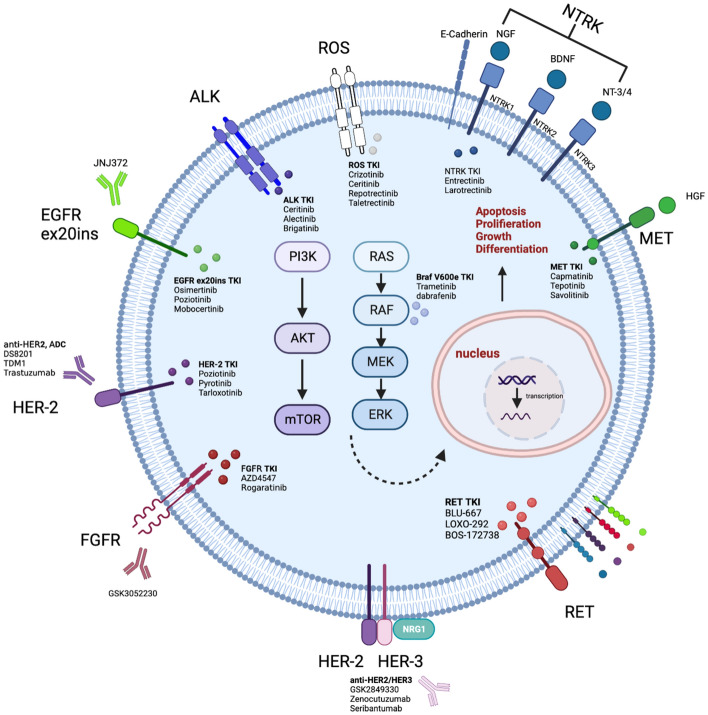
Simplified overview of the molecular pathways affected by novel emerging rare targets in NSCLC and agents in clinical development. These pathways are physiologically activated by the interaction between circulating growth factors (colored circles) and transmembrane receptors (colored sticks crossing the cell membrane), leading to the downstream activation of intracellular proteins (colored ovoids) that promote cell proliferation, increased aggressiveness, or immune escape. Investigational agents that can inhibit specific pathways are also reported, along with their targeted molecules or interactions, when available. For more details, please refer to the appropriate paragraphs.

With the development of detection technology, several oncologic drivers have been identified, and corresponding agents have been developed by researchers in the last two decades. The FDA has approved multiple targeted agents for patients who have alternative oncologic drivers, such as *EGFR*, *ALK*, *ROS1*, *BRAF*, *Met*, *Ret, FGFR* and *NTRK*. Additionally, the development of new-generation TKIs and immunotherapeutic agents has further improved treatment outcomes, with some patients achieving long-term survival benefits. Most clinical studies have demonstrated that patients harboring some type of oncologic driver using targeted therapy can achieve better survival benefits than those receiving chemotherapy or immunotherapy. This has led to a significant shift in the management of NSCLC, with targeted therapy becoming the preferred first-line treatment option for patients with actionable mutations and guiding subsequent researchers and practitioners (Fig. 1).

Currently, an increasing number of rare oncogenic drivers and activating mutations have been discovered and reported. Does it make sense to study corresponding TKIs and related targeted therapies? According to global cancer statistics, lung cancer is the second largest tumor in the world with a large number of patients, and lung cancer-related deaths remain the leading cause of cancer-related deaths worldwide. Lung cancer is still the leading cause of cancer-related death worldwide, and its incidence and mortality rates are 3- to fourfold greater in developed countries than in developing countries, mainly due to the tobacco epidemic in developing countries. Additionally, the incidence and mortality rates of lung cancer have shown an increasing trend not only in developed countries but also in developing countries around the world. Thus, it might be meaningful for researchers to find novel oncologic drivers and develop innovative drugs, especially in developed and developing regions.

Second, there is high funding for pan-cancer next-generation sequencing (NGS)-based gene panel testing, such as the 425-gene panel. For developing areas, not everyone has the economic conditions to complete it. Therefore, with common oncologic driver alterations, such as EGFR, and some rare oncologic driver alterations, such as *RET*, *ALK*, *ROS* and *NTRK*, we suggest that practitioners should include these in a new panel. This strategy can decrease the economic pressure on patients. Otherwise, practitioners also can use IHC, FISH, and polymerase chain reaction (PCR) to target specific gene alterations, such as MET with high GCN or HER-2 with IHC3 + , and identify corresponding patients such as patients with locally advanced and advanced lung cancer who may benefit from targeted therapies.

Third, new drugs may not lead to a relatively broad spectrum and may also generate drug resistance genes. Drug resistance can occur, such as generation of *EGFR T790m* or *RET*-related solvent front mutations. Thus, patients need to reconsider the use of a pan-cancer gene panel. Furthermore, researchers should focus on these rare mutations and develop related new agents.

Then, researchers should develop new agents that should not only cover common activating mutations, such as *EGFR L858R* and exon 19 deletion but also cover rare activating mutations, such as exon 20 insertion. Although some alterations in oncogenic drivers, such as HER-2 IHC + , IHC2 + , and MET amplification with low GCN and *FGFR* alternation, have shown limited effectiveness in clinical trials. More subgroups should be explored like IHC2 + and FISH + in gastric carcinoma and breast cancer. Moreover, researchers should develop next-generation drugs to provide significant survival benefits. In addition, researchers also can explore new regimens like TKIs plus chemotherapy and TKIs plus monoclonal antibodies or ADCs, and corresponding clinical trials should also be developed. However, clinical trials and monitoring of side effects are necessary, as some targeted agents may cause severe side effects, such as cardiac toxicity with trastuzumab. Therefore, patients should receive baseline examinations before receiving TKIs or other targeted agents. Although most of target agents are relatively safe, monitoring of side effects is necessary for each patient.

Finally, targeted therapy is now the preferred treatment option for NSCLC patients with actionable mutations. To achieve a breakthrough in molecular targeted therapy for advanced NSCLC and improve patient outcomes, researchers must continuously work toward developing new testing methods, therapies, and combination treatments. Additionally, gaining a deeper understanding of resistance mechanisms is essential for overcoming treatment resistance and improving patient response rates. These efforts could potentially lead to a paradigm shift in the treatment of locally advanced or advanced NSCLC and offer patients a more positive prognosis. In addition, researchers also cannot ignore the related side effects of targeted therapy. Therefore, each patient can achieve optimal survival benefits.

## Data Availability

All data generated or analysed during this study are included in this published article.
